# Adipokinetic hormone signaling determines dietary fatty acid preference through maintenance of hemolymph fatty acid composition in the cricket *Gryllus bimaculatus*

**DOI:** 10.1038/s41598-018-22987-2

**Published:** 2018-03-16

**Authors:** Keisuke Fukumura, Takahiro Konuma, Yusuke Tsukamoto, Shinji Nagata

**Affiliations:** Department of Integrated Biosciences, Graduate School of Frontier Sciences, The University of Tokyo, Chiba, 277-8562 Japan

## Abstract

Adipokinetic hormone (AKH), an analog of mammalian glucagon, functions in supplying the required energy by releasing lipids and carbohydrates from the fat body into the hemolymph. Our previous study showed that *AKH receptor* (*AKHR*) knockdown in the two-spotted cricket *Gryllus bimaculatus* decreased hemolymph lipid levels and increased its feeding frequency. To reveal underlying mechanisms by which AKH signaling modulates lipid homeostasis, we analyzed the fatty acid composition as the lipid structure in the crickets. AKH administration significantly increased the proportion of unsaturated fatty acids (USFAs) to total fatty acids with decrease of the saturated fatty acids (SFAs) in hemolymph, while these proportions were inversely changed in RNA interference-mediated *AKHR*-knockdowned (*AKHR*^RNAi^) crickets. Interestingly, knockdown of *hormone-sensitive lipase* (*Hsl*) by RNAi (*Hsl*^RNAi^) affected the proportion of USFAs and SFAs in a similar manner to that observed in *AKHR*^RNAi^ crickets. AKH administration in *Hsl*^RNAi^ crickets did not change hemolymph fatty acid composition, indicating that AKH signaling critically altered fatty acid composition in the hemolymph through Hsl. In addition, a choice assay revealed that *AKHR*^RNAi^ significantly increases the preference of USFAs. These data indicate that hemolymph lipid level and composition were modulated by AKH signaling with a complementary feeding behavior toward USFAs.

## Introduction

Insect adipose tissue, the fat body, plays a crucial role in lipid biosynthesis, metabolism, and energy storage. Lipids and carbohydrates stored in the fat body are used as energy sources in various situations, such as for a long flight by migratory insects as observed in the desert locust *Schistocerca gregaria* and the migratory locust *Locusta migratoria*^1,2^. In most insects, energy is acquired by consuming dietary nutrients by feeding and by catabolizing lipids and glycogen in the fat body, which are then mobilized into the circulating system (hemolymph) similar to that observed in other animals^3,4^. Such energy demands, including lipid mobilization, are under endocrine control involving adipokinetic hormone (AKH), an analog of mammalian glucagon^5,6^. Mobilization of lipids from the fat body into the hemolymph involves specific fat body lipases that hydrolyze triacylglycerol (TAG) to diacylglycerol (DAG) and fatty acids^7,8^. Several studies indicate that AKH promotes the conversion of TAG to DAG during mobilization of lipids^7,9,10^. Therefore, the turnover of TAG, DAG, and fatty acids is thought to be strongly associated with AKH-regulated energy homeostasis in insects^3,11^. To date, identifications of over 60 AKH precursors realize that AKH is the highly conserved peptide hormone in the arthropods^5,12^. Moreover, in addition to their original function to mobilize carbohydrates and lipids from the fat body into the hemolymph, several different aspects of AKHs have been demonstrated to play physiological roles ranging from oxidative stress resistance, heartbeat control, and responses in immunity against fed substances^13–16^.

Manipulating the transcriptional level of AKH receptor (AKHR) has demonstrated AKH contribution to diverse physiological and biological processes. In the two-spotted cricket *G. bimaculatus*, *AKHR* knockdown by RNA interference (RNAi) increases feeding frequency and food intake^17^. In *Drosophila melanogaster*, loss-of-function in AKHR results in starvation resistance^10^. Moreover, AKHR-mediated mobilization of carbohydrates and lipids has been observed in various insects, including the yellow fever mosquito *Aedes aegypti*, the blood-sucking insect *Rhodnius prolixus*, and the cockroach *Blattella germanica*^18–20^. The phenotypic changes induced by *AKHR* manipulation at the transcriptional level have highlighted the importance of AKH signaling in altering lipid and carbohydrate levels accompanied by regulating feeding behavior of insects.

Recent studies have demonstrated modification of the fatty acid composition in response to changes in the physiological conditions of several insect species^21,22^. Changes in the fatty acid composition of the codling moth *Cydia pomonella* reinforce maintenance of cellular membrane structures and stored lipid levels, even under unfavorable conditions such as low temperature^23^. Therefore, lipid composition can be a crucial factor for responding to changes in environmental conditions.

The lipid composition in tissues or the hemolymph is influenced by the state of lipid metabolism. In particular, lipid composition is linked to the degree of flux in lipid usage and synthesis and the corresponding status of enzymatic activities in lipogenesis and lipolysis. In addition, the structural characteristics of lipids in the carbon chain length and the saturation degree of fatty acids indicate a temporal influence of lipid metabolism on the nutrient state. Therefore, AKH signaling must modulate lipid biosynthesis and metabolism, eventually causing changes in the fatty acid composition, according to the nutritional state following feeding behavior.

Along with alterations in the hemolymph lipid levels under different physiological and biological conditions, the previous study has demonstrated that the lipid biochemical composition in the fat body changes during adult development in the cricket^24^. Furthermore, AKH signaling has been shown to affect the fatty acid composition of the fat body in the tenebrionid beetle *Zophobas atratus*^25^. Taken together, these findings suggest that AKH signaling is involved in both qualitative and quantitative aspects of lipid homeostasis in insects. However, the relationship between AKH signaling and feeding behavior and the relationship between lipid metabolism and feeding behavior in insects remains unclear.

In the present study, we explored the contribution of AKH signaling in maintaining the hemolymph fatty acid composition to better understanding of the underlying regulatory mechanisms of insect feeding behavior. We also demonstrate that lipid composition changes are caused by differences in the transcriptional levels of lipogenic and lipolytic enzymes, which in turn is regulated by AKH signaling. In addition, we demonstrate that hemolymph lipid changes resulted in the modification of preferential feeding behaviors to compensate for the imbalanced lipid component.

## Results

### Effects of AKH injection on lipid mobilization

**Figure 1:**
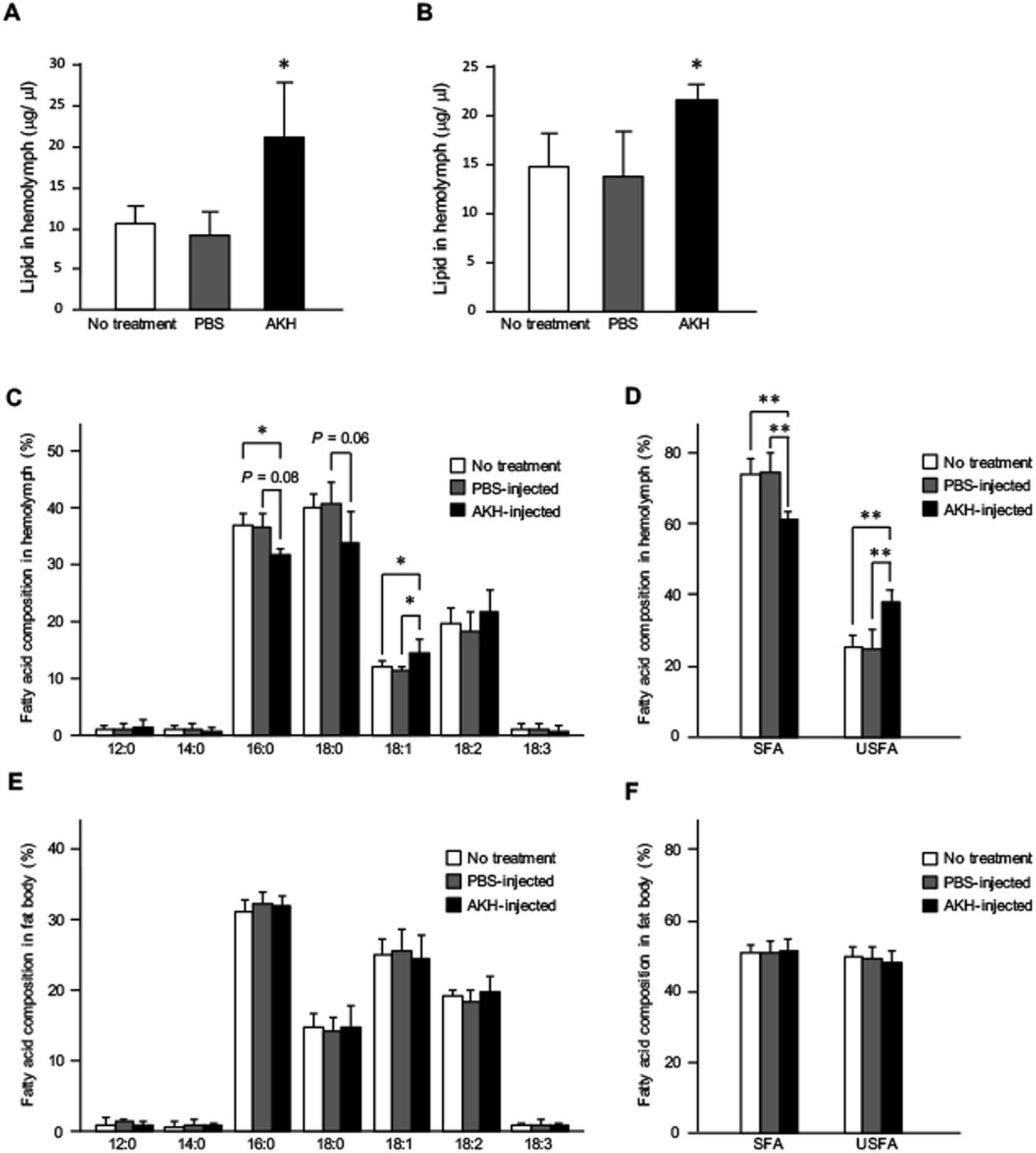
Effects of AKH injection on lipid levels and lipid composition in crickets. Hemolymph lipid levels in AKH-injected adult crickets (**A**) and fifth instar cricket nymphs (**B**). The lipid levels after AKH injection were compared with those of non-treated crickets. Also, the lipid levels by AKH injection were compared with those of PBS-injected crickets after 90 min. Fatty acid composition in the hemolymph of fifth instar cricket nymphs (**C**) and the proportions of SFAs and USFAs to total fatty acids (**D**). Fatty acid composition in the fat body of fifth instar cricket nymphs (**E**) and the proportions of SFAs and USFAs to total fatty acids in the hemolymph (**F**). Values are expressed as means ± SD (A; n = 5, B; n = 5–6, C; n = 5, D; n = 5, E; n = 7, F; n = 7, *P < 0.05 for Tukey’s post hoc test).

We first confirmed the effects of AKH injection on adult crickets, which triggered a significant increase in hemolymph lipid level compared with those of no treatment, while the lipid level of PBS-injected crickets did not alter (Fig. 1A). We also examined the effects of AKH injection on lipid mobilization in cricket nymphs to exclude the influence of reproduction. Injection of AKH into fifth instar cricket nymphs increased hemolymph lipid level by 26.1% compared with lipid level of non-treated crickets, while the lipid level of PBS-injected crickets did not alter (Fig. 1B), consistent with the data using adult crickets (Fig. 1A). Hereafter, we thus used the cricket nymphs for all studies.

### Effects of AKH injection on fatty acid composition

To investigate these changes in more detail, we analyzed the hemolymph lipid composition of DAG- and TAG-derived fatty acids. In the present analyses of fatty acid composition using HPLC, we analyzed most abundant seven fatty acids, which were observed in the hemolymph and fat body of cricket nymphs; lauric acid (12:0), myristic acid (14:0), palmitic acid (16:0), stearic acid (18:0), oleic acid (18:1), linoleic acid (18:2), and linolenic acid (18:3). AKH injection decreased the proportions of palmitic (16:0) and stearic acids (18:0) to total fatty acids, whereas the proportions of oleic acid (18:1) was significantly increased in the hemolymph (Fig. 1C). In contrast, fatty acids derived from the fat body in the AKH-injected crickets showed negligible changes in their composition (Fig. 1E). Incidentally, AKH injection significantly decreased the proportion of saturated fatty acids (SFAs; 16:0 and 18:0) to the total fatty acids and increased the proportion of unsaturated fatty acids (USFAs; 18:1, 18:2, and 18:3) in the hemolymph, whereas these proportions in the fat body were unchanged (Fig. 1D and F).

### Effects of *AKHR* knockdown on fatty acid composition

**Figure 2:**
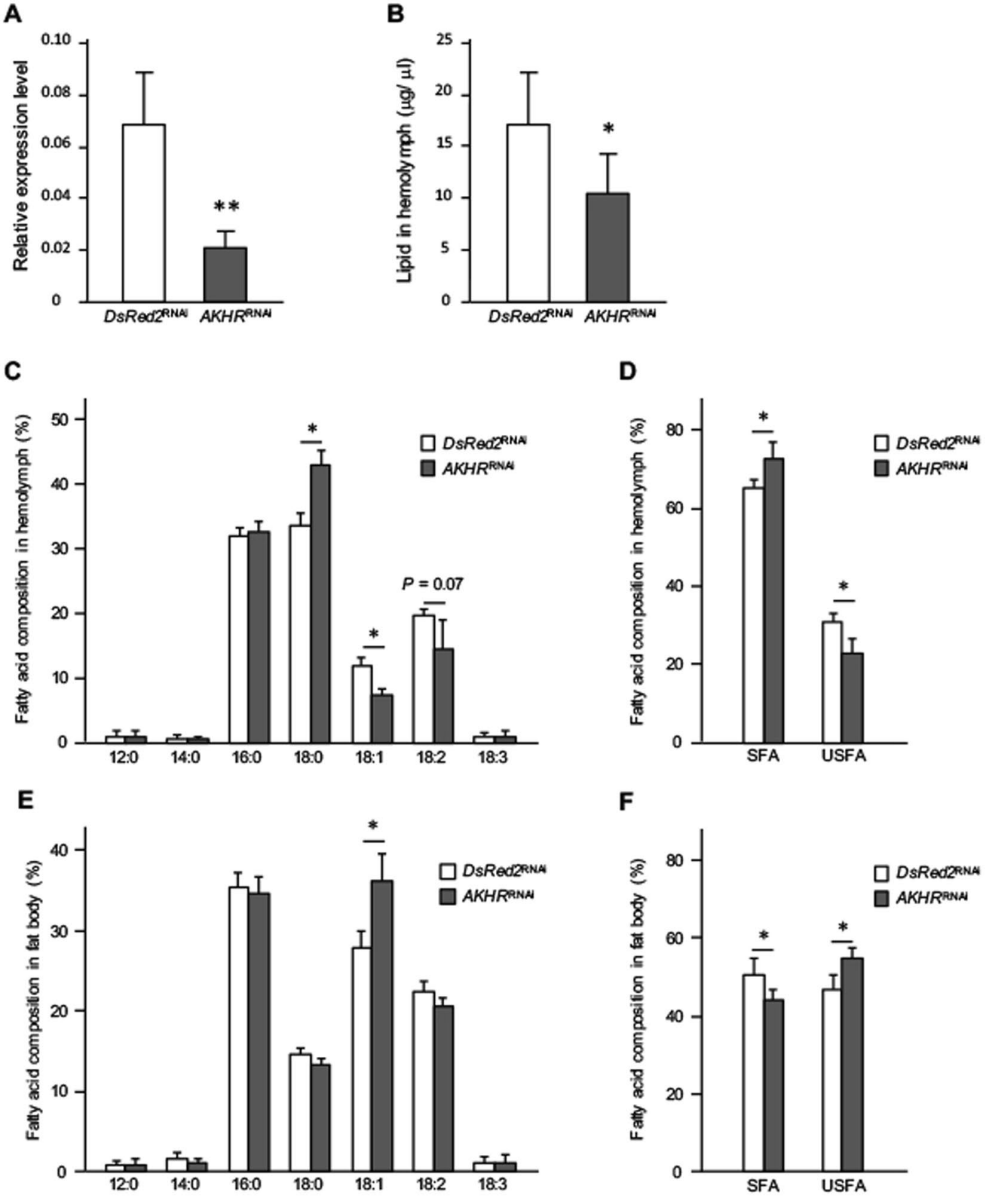
Effects of RNAi-mediated *AKHR* knockdown on lipid levels and lipid composition. The *AKHR*^RNAi^ crickets were compared with the experimental control crickets treated with dsRNA encoding *DsRed2*. The transcriptional level of *AKHR* in the fat body compared with transcript levels of elongation factor (**A**). Hemolymph lipid levels in *AKHR*^RNAi^ crickets (**B**). Fatty acid composition in the hemolymph of *AKHR*^RNAi^ crickets (**C**), and the proportions of SFAs and USFAs to total fatty acids (**D**). Fatty acid composition in the fat body of *AKHR*^RNAi^ crickets (**E**), and the proportions of SFAs and total USFAs (**F**). Values are expressed as means ± SD (A; n = 8, B; n = 6, C; n = 5, *D*; n = 5, E; n = 5, F; n = 5, **P < 0.01 and *P < 0.05 for Student’s *t*-test).

Because the fatty acid composition in the hemolymph was altered by AKH injection, we further analyzed the fatty acid composition by disrupting AKH signaling using cricket after treatment of *AKHR* RNAi (*AKHR*^RNAi^). The effects of *AKHR* knockdown were confirmed by qRT-PCR (Fig. 2A). Similar to the previous observation^17^, hemolymph lipid levels were significantly decreased in *AKHR*^RNAi^ crickets, whereas the control crickets (*DsRed2*^RNAi^) did not alter the levels (Fig. 2B). We then analyzed the fatty acid composition in the hemolymph and fat body of *AKHR*^RNAi^ crickets. In the hemolymph of *AKHR*^RNAi^ crickets, the proportion of stearic acid (18:0) to total fatty acids was significantly increased, while the proportions of oleic (18:1) and linoleic acid (18:2) were decreased (Fig. 2C). In contrast, in the fat body, the proportion of oleic acid (18:1) was significantly increased (Fig. 2E). Totally, knockdown of *AKHR* significantly increased the proportion of SFAs to total fatty acids and decreased that of USFAs in the hemolymph (Fig. 2D), whereas the opposing changes in those proportions were observed in the fat body (Fig. 2F).

### Effects of *AKHR* knockdown on lipid metabolism-related genes

**Figure 3:**
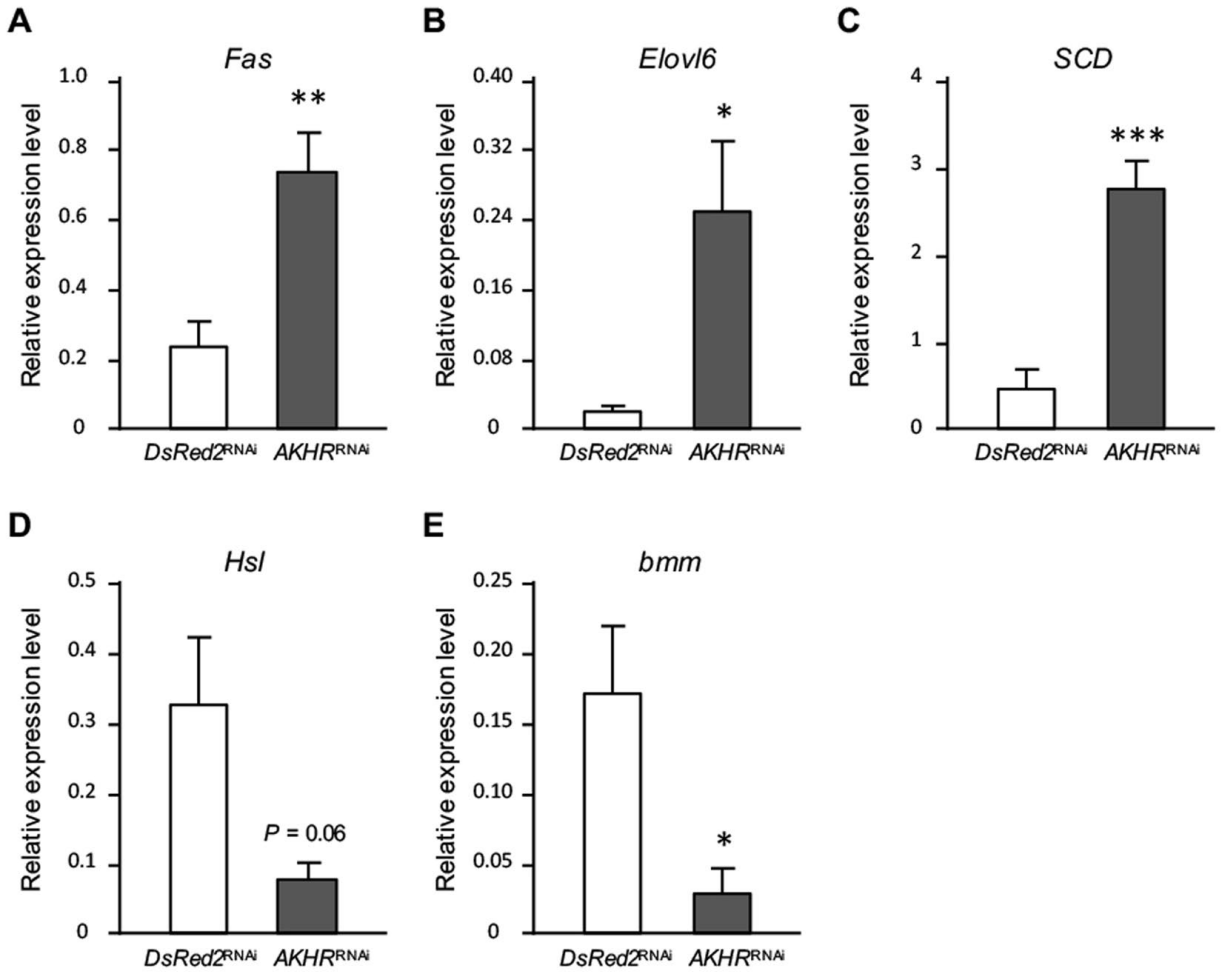
Effects of AKH signaling on the expression of genes involved in lipid metabolism. The relative transcriptional levels of lipogenic and lipolytic genes compared with transcriptional levels of elongation factor in *AKHR*^RNAi^ crickets (**A**–**E**). The experimental control was performed using crickets treated with dsRNA encoding *DsRed2*. Values are expressed as means ± SD (n = 5, ***P < 0.001 and *P < 0.05 for Student’s *t-*test).

As observed above, manipulating AKH signaling drastically changed the fatty acid composition in the hemolymph and fat body. To determine whether the lipid biosynthetic pathway in the fat body mediates AKH signaling-induced changes in hemolymph fatty acid composition, we analyzed the transcriptional levels of genes associated with lipid metabolism in the fat body of *AKHR*^RNAi^ crickets. We measured the transcriptional levels of three genes encoding enzymes which critically contribute to the fatty acid composition: *fatty acid synthase* (*Fas*), *stearoyl-CoA 9 desaturase* (*SCD*), and *elongation of very long chain fatty acids protein 6* (*Elovl6*). In addition, we also measured the transcriptional levels of two lipases involved in fatty acid mobilization from DAGs; *hormone-sensitive lipase* (*Hsl*) and *brummer* (*bmm*). qRT-PCR showed that the transcriptional levels of *Fas*, *SCD*, and *Elovl6* were significantly increased in *AKHR*^RNAi^ crickets compared with those in control dsRNA-treated crickets (Fig. 3A–C). In contrast, the transcriptional levels of *Hsl* and *bmm* were decreased in *AKHR*^RNAi^ crickets compared with those in the control crickets (Fig. 3D and E). These results showed that AKH signaling modulated the flux of lipids by controlling both lipid biosynthesis in the fat body and lipid mobilization from the fat body into the hemolymph at the transcriptional level.

### Involvement of Hsl in the AKH-mediated fatty acid flux

**Figure 4:**
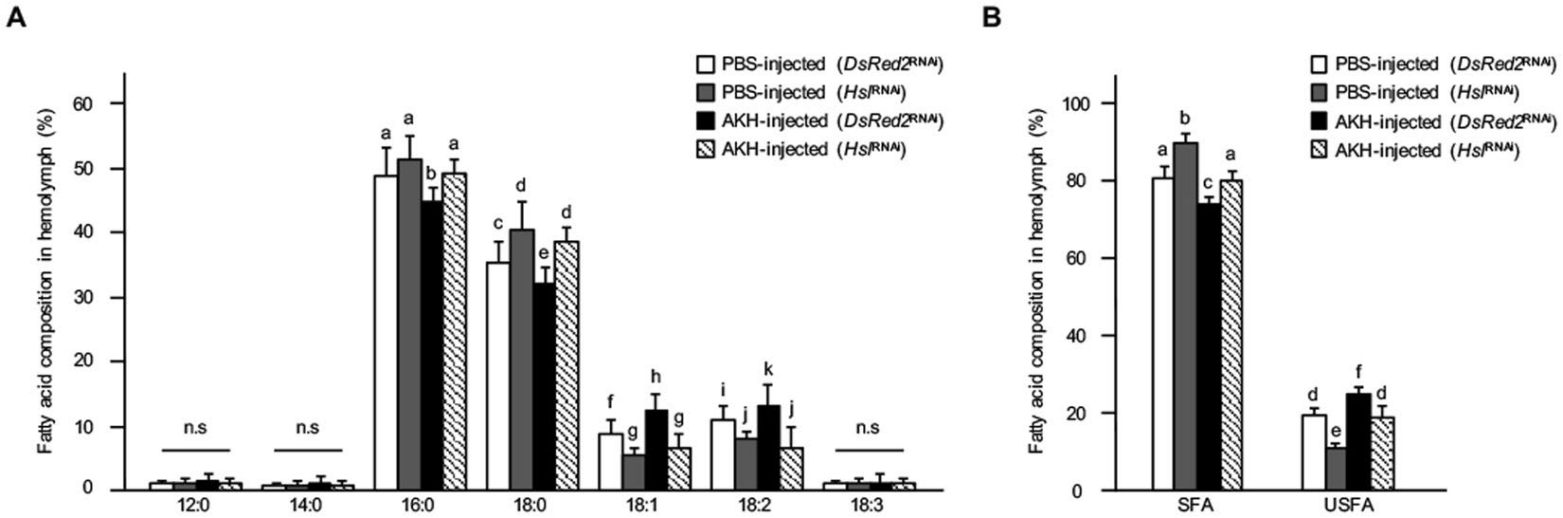
Effects of RNAi-mediated *Hsl* knockdown on fatty acid composition. The fatty acid composition in the hemolymph of *Hsl*^RNAi^ crickets with or without AKH injection (**A**), and the proportions of SFAs and USFAs to total fatty acids (**B**). Values are expressed as means ± SD (A and B; n = 7). The statistical analyses were performed within the same structure (**A**) or within same structural group (**B**). Different letters indicate significant differences over Tukey’s post hoc test (*P* < 0.05), n.s. indicates not significant.

Because AKH injection and *AKHR* knockdown demonstrated that AKH signaling influenced the composition of hemolymph fatty acids, we next investigated the relationship between lipase activity and the hemolymph fatty acid composition. Similar to mammalian lipid metabolism in which glucagon activates Hsl activity, AKH regulates Hsl function in *D. melanogaster*^26,27^. It appears that AKH likewise activates cricket Hsl. We therefore analyzed the fatty acid composition in the hemolymph of crickets following *Hsl* knockdown (*Hsl*^RNAi^). The proportion of USFAs was significantly decreased in the hemolymph of *Hsl*^RNAi^ crickets accompanied by an increase in the proportion of SFAs to total fatty acids, while little difference was observed in the hemolymph proportions of SFAs and USFAs after injection of AKH into *Hsl*^RNAi^ crickets compared with those in *DsRed2*^RNAi^ crickets (Fig. 4A and B). These results indicated that Hsl was involved in the maintenance of the fatty acid flux via AKH signaling, possibly with Hsl substrate preference for USFAs as reported previously^28^.

### Effects of *AKHR* knockdown on the feeding preference for fatty acids

**Figure 5:**
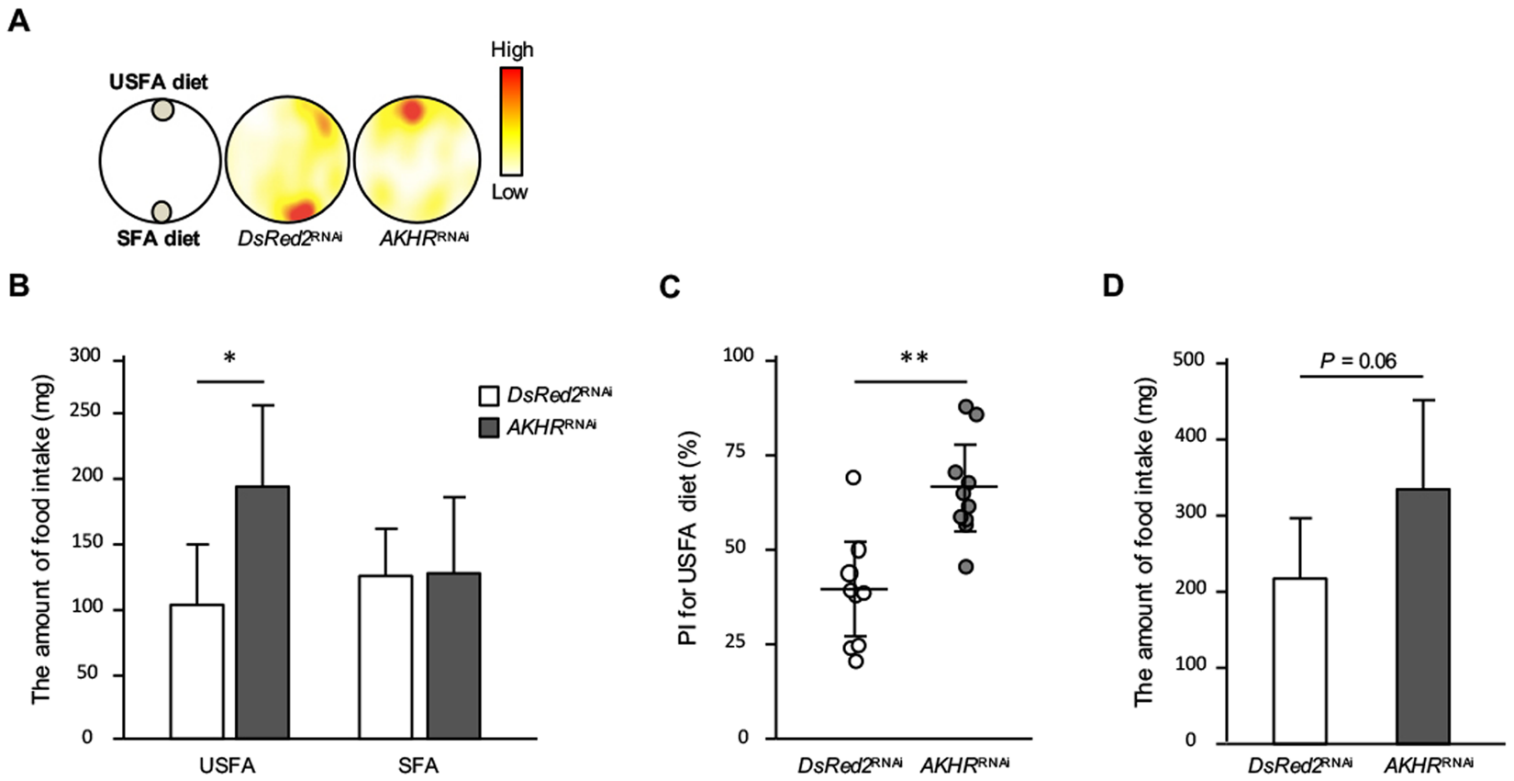
Effects of RNAi-mediated *AKHR* knockdown on the feeding preference. Representative feeding behaviors for 9 h (**A**). Colors represent the duration of time a cricket spent within a particular area with thicker red indicating longer periods of time. The amount of food intake from the USFA or SFA diets (**B**). PI for the USFA diet (**C**). Total food intake of SFA and USFA diets (**D**). Values are expressed as means ± SD (B,C, and D; n = 9, **P < 0.01 and *P < 0.05 for Student’s *t-*test).

Since *AKHR*^RNAi^ reduced the proportion of hemolymph USFAs, we examined the selective feeding behavior for dietary lipid contents in *AKHR*^RNAi^ crickets using a two-choice assay with two diet blocks containing different fatty acids: an USFA-included diet (USFA diet) and a SFA-included diet (SFA diet). Time-lapse recording of the cricket movement showed that *AKHR*^RNAi^ crickets exhibited increased feeding frequency on USFA diet compared with that by *DsRed2*^RNAi^ crickets (Fig. 5A). Furthermore, the amount of intake from the SFA diet in the *AKHR*^RNAi^ crickets was indistinguishable from that of control crickets, whereas the amount of USFA diet intake was significantly increased in *AKHR*^RNAi^ crickets (Fig. 5B and C). Also, overall food intake by *AKHR*^RNAi^ crickets was increased (Fig. 5D) as previous report^17^. These results indicated that *AKHR*^RNAi^ changed the feeding preference for fatty acids, which apparently complement the imbalanced hemolymph lipid component.

## Discussion

The originally characterized function of AKH is mobilization of lipids from storage in the fat body for large energy demands such as long flight during migration^1,2^. Therefore, most studies on AKH regulation have been performed using adult insects such as migratory locusts^6^. The involvement of AKH in lipid metabolism not only for acute energy demands but also in the function of nutritional homeostasis has been highlighted recently, and has shown AKH to be an insect analog of mammalian glucagon^26,29^. These findings indicate that AKH is crucial for maintaining lipid and carbohydrate levels in the insect body throughout its life cycle. In the present study, we confirmed that the effects of AKH on lipid mobilization in pre-adult *G. bimaculatus* are comparable to those reported for adult crickets^17,30^. Our results using nymphal crickets also indicated that AKH can contribute to lipid homeostasis in the absence of adult specific events such as reproduction required for additional energy demands (Figs 1B, 2A and B).

The current study illustrates that fatty acid composition is correlated with lipid biosynthesis and metabolism, which is influenced by nutritional conditions in the crickets. Indeed, previous studies using several insect species have demonstrated that fatty acid composition is regulated by physiological and biochemical factors, including dietary conditions and surrounding temperature^21–23^. Therefore, the composition of fatty acids is a consequence of the metabolic and nutritional states. Unfortunately, except for a recent report using the beetle *Zophobas atratus*^25^, few studies have reported whether the metabolic regulation involved in AKH signaling can control fatty acid composition. In the present study, AKH stimulation increased the proportion of USFAs composed of oleic acid (18:1), linoleic acid (18:2) and linolenic acid (18:3) in the hemolymph of cricket nymphs (Fig. 1C and D). This finding is consistent with that reported in the beetle study^25^. In contrast, the proportion of USFAs to total fatty acids decreased in the hemolymph of *AKHR*^RNAi^ crickets (Fig. 2C and D). These opposing data might be due to differences in the nutritional states. In the AKH-injected crickets, fatty acids in the fat body that respond to AKH stimulation for acute energy demands were mobilized even under normal conditions. In contrast, *AKHR*^RNAi^ crickets were in a state of chronic AKH signaling deficiency; thus it was difficult for those insects to maintain basal hemolymph lipid levels and may have induced changes in the mechanisms underlying lipid homeostasis maintenance. Data in Fig. 2 implied that hemolymph USFAs were apparently replaced by SFAs. In fact, allocation of more USFAs after AKH stimulation via hemolymph to the organs such as muscle might have physiologically benefits to produce ATP easier via β-oxidation as an energy source to supply energy demand for a long flight. This speculation is consistent with the fact that USFAs is rapidly catalyzed by β-oxidation compared with SFAs^31^.

We also demonstrated the role of a lipase in the fatty acid composition changes observed in the hemolymph. It has been reported so far that insects have at least two lipolytic systems that are mediated by AKH signaling and non-AKH signaling, which includes bmm activity^10,32^. In *D. melanogaster*, they function differently based on the nutritional state, including the trans-state as they shift from hungry to starved^10^. Based on the different composition of fatty acids in the hemolymph after AKH injection, it can be concluded that AKH injection activated Hsl in the fat body. Hsl activation might cause a biased-preference in the enzymatic substrate, which would eventually change the fatty acid composition in the hemolymph. This is also consistent with previous reports showing that Hsl in the rat *Rattus norvegicus* and the Antarctic fish *Trematomus newnesi* has characteristic catabolism in substrate specificity^33,34^. This speculation agrees with our data showing an increase in the proportion of SFAs with the simultaneous reduction of USFAs in the hemolymph of *Hsl*-knockdown crickets (Fig. 4). Moreover, AKH injection in *Hsl*^RNAi^ crickets did not affect the proportions of SFAs and USFAs to total fatty acids in the hemolymph, indicating that AKH signaling influences the hemolymph fatty acid composition via Hsl activity in the fat body.

In *AKHR*^RNAi^ crickets, significant changes were observed in the transcriptional levels of lipogenic and lipolytic genes (Fig. 3), indicating that the decreased level of hemolymph lipids and accumulation of lipid level in the fat body are involved in AKH signaling^17,30^. In the present study, AKH-injected crickets showed an acute lipid mobilization response within 90 minutes, indicating that such acute transcriptional changes in the lipogenic and lipolytic genes likely have little role in affecting changes in the nutritional state of these crickets. In contrast, *AKHR*^RNAi^ crickets showed a chronic deficiency in AKH signaling, indicating that the transcriptional levels of the lipogenic and lipolytic genes in the fat body of these nymphs were altered. These data revealed that AKH plays roles in both acute and chronic lipid mobilization, although the transcriptional contribution to lipogenesis and lipolysis are different.

Similar to other animals, insects need to discriminate and select the appropriate nutrition from their diet to complete various life events including development and reproduction. In *D. melanogaster*, the feeding preference for dietary fatty acids was shifted according to nutritional demands during development^35^. Because hemolymph components, such as lipids, are the critical factors that reflect the individual nutritional states, feeding behaviors to seek the appropriate nutrition must be affected by the nutritional state of the hemolymph as observed in *Locust migratoria*^36^. In this study, *AKHR*^RNAi^ decreased the proportion of USFAs in the hemolymph and increased the amount of food intake of a USFA diet (Figs 2D and 5). This result indicated that *AKHR*^RNAi^ crickets discriminated the different chemical structures of fatty acids and selectively ingested the USFA diet to compensate for the nutritional loss in the hemolymph. However, it remains unknown whether increased feeding motivation for USFAs is regulated by AKH signaling directly at a molecular level.

In summary, we demonstrated that AKH signaling mobilizes lipid from the fat body with changes of fatty acid composition in the hemolymph of cricket nymphs. The mobilized lipids by AKH stimulation might be composed of more USFAs such as oleic acid (18:1) in the hemolymph. In the case of activated AKH signaling, those changed composition of fatty acids is possibly mediated by regulation of lipid metabolic gene expression and Hsl activation. In addition, AKH signaling can modulate the feeding preference for SFAs or USFAs directly or indirectly, possibly according to the required nutrients in various biological processes. Understanding these behavioral changes will help further elucidate the mechanisms linking lipid homeostasis and insect feeding behavior.

## Methods

### Chemicals and reagents

Palmitic, stearic, oleic and linoleic acids were purchased from Nacalai tesque (Kyoto, Japan). Cellulose, dextrin and casein powders were purchased from CLEA Japan (Tokyo, Japan).

### Insects

Crickets were reared in plastic containers (55 × 39 × 31 cm) at 28 ± 1 °C under long-day lighting conditions (16-h light/8-h dark cycle). Fifth instar cricket nymphs had free access to water and were fed *ad libitum* a standard diet of rabbit food ORC4 (Oriental Yeast, Tokyo, Japan) and cat food at a 3:1 ratio.

### Preparation and injection of AKH

*Gryllus bimaculatus* AKH (p-QVNFSTGW-NH_2_) was chemically synthesized as described previously^17,30^. AKH was injected into the hemolymph through the abdomen with 20 pmol AKH in 5 μl of Phosphate-buffered saline (PBS) or 5 μl of PBS injected into each cricket. To measure hemolymph lipids, 5 μl of hemolymph was collected from crickets prior to injection (0 min) and 90 min after injection.

### Lipid extraction from the hemolymph and fat body

Lipids were extracted as described by Lorenz with minor modification^17,30,37^. Briefly, 5 μl of hemolymph or the fat body surrounding the testis were collected in centrifuge tubes containing 20 mg of sodium sulfate and 200 μl of 75% methanol in water. Next, the hemolymph and fat body were homogenized in 600 μl chloroform: methanol (1:1) by sonication and centrifuged at 15,000 × *g* at 4 °C for 10 min. The supernatant was then transferred to a new tube, vortexed, and centrifuged at 15,000 × *g* at 4 °C for 10 min after adding 500 μl of chloroform and 300 μl of 1 M NaCl. The organic layer containing lipids was dried under vacuum and centrifugation. The resulting lipid fraction was then used for lipid quantification.

### Measurement of lipid

The lipids extracted from the hemolymph and fat body were quantified using the previously reported sulfo-phospho-vanillin method^17,30,38^. The extracted lipid fractions in chloroform/methanol (1 μl) were mixed with 50 μl of sulfuric acid and heated at 100 °C for 10 min. After cooling the solution to room temperature, 500 μl of vanillin reagent (0.2% vanillin in 67% ortho-phosphoric acid) was added. The resulting samples were measured at 540 nm in a spectrophotometer. Cholesterol (Sigma-Aldrich Japan, Tokyo, Japan) was used as a standard. The lipids from the hemolymph and the fat body were quantified as DAG and TAG, respectively.

### Fatty acid composition analysis by high-performance liquid chromatography

An HPLC (Jasco SC-802, PU-880, UV-875; Jasco Inc., Tokyo, Japan) equipped with a fluorescence detector (excitation at 365 nm, emission at 412 nm; RF-20A; Shimadzu Co., Kyoto, Japan) was used for analyzing the fatty acid composition. HPLC was performed using a reversed-phase column (PEGASIL C_8_ SP100, 4.6ϕ × 250 mm; Senshu Scientific Co., Tokyo, Japan) at 40 °C in an oven (865-CO; JASCO, Tokyo, Japan). After saponification of the lipid fraction, fatty acids were identified and quantified by comparing retention times with those of commercially available standard fatty acids. The mobile phase was acetonitrile: water (80:20 [v/v]) at a flow rate of 1.5 ml/min. Fluorescent 9-anthryldiazomethane (ADAM) ester derivatives of fatty acids were prepared as described previously^39^. Briefly, the lipid fraction was saponified by incubating with 200 μl of 1 M KOH in 90% methanol at 90 °C for 1 h. The reaction mixture was then neutralized by adding 6 μl of HCl. The resulting free fatty acids were extracted using hexane and dried prior to being resuspended in 30 μl chloroform: methanol (1:1). The fatty acid solutions (5 μl) were incubated in the dark with 50 μl of 0.1% ADAM in acetone: methanol (1:9) for 1 h at room temperature. The mixture was then subjected to HPLC.

### Identification of genes involved in lipid metabolism

The genes involved in lipid metabolism were identified using Hiseq2000 (Illumina Inc., San Diego, CA, USA) sequencing data derived from *G. bimaculatus* subesophageal ganglion and fat body^40^. These data were subject to *de novo* assembly using the CLC genomics workbench (CLC bio, Aarhus, Denmark). Genes encoding *fatty acid synthase* (*Fas*) (KU254604.1), *elongation of very long chain fatty acids protein 6* (*Elovl6*) (KU254604.1), *stearoyl-CoA 9 desaturase* (*SCD*) (KU296958.1), *hormone-sensitive lipase* (*Hsl*) (KU254602.1) and *brummer* (*bmm*) (KU254603.1) were identified based on sequence homology with genes from the other insect species: *Fas1* (ACL82985.2), *Elovl6* (JAG70648.1), *SCD1* (AF338465.1), *Hsl ortholog* (AAM68400.1), and *brummer* (NP_001163445.1).

### Quantitative RT-PCR

Total RNA was isolated from tissues with TRIzol (Invitrogen, Carlsbad, CA, USA) according to the manufacture’s protocol. The extracted RNA was treated with RQ DNase I (Promega, Madison, WI). The extracted RNA (100 ng) was reverse-transcribed using ReverTra Ace (Toyobo, Osaka, Japan) and an oligo-(dT)_20_ primer. The resulting cDNA was used as a template for quantitative RT-PCR (qRT-PCR). qRT-PCR was performed using SYBR Premix Ex Taq II (Tli RNaseH Plus; TaKaRa, Shiga, Japan) on a Thermal Cycler Dice Real Time System TP850 (TaKaRa, Shiga, Japan). All reactions were performed in duplicate, and reproducibility was confirmed using different sample sets. Relative mRNA levels were calculated using the comparative Ct method. All results were standardized using elongation factor as an experimental control since no statistically differences were observed among data obtained with two other reference genes (*rpl32* and *β-actin*). Sequences of the primers are as follows: *SCD*-Fw 5′-TGGTCAAATGCCTGGTTTG-3′, *SCD*-Rv 5′-GCACTGTTCACCAGCCAAG-3′, *Fas*-Fw 5′-GCTTGCCCCACTTTCATGC-3′, *Fas*-Rv 5′-GCAAGGGGAGCTTTTTCCG-3′, *Elovl6*-Fw 5′-GCTGCCAGAGCTGTATTCCA-3′, *Elovl6*-Rv 5′-AAAGTCGGCCACGGTTTCAA-3′, *Hsl*-Fw 5′-TCTGTGCCTCATGGATCCCT-3′, *Hsl*-Rv 5′-TGAGGAGTCGGGCAAGCATA-3′, *bmm*-Fw 5′-TGGGCATGTCAGTTCCTTGT-3′, *bmm*-Rv 5′-AGAGAGCTCCCGGGACAAAT-3′, *rpl32*-Fw 5′-CAAACTGGAGGAAACCGAAA-3′, *rpl32*-Rv 5′-ATCAACCTTTGGCCCTTGA-3′, *elongatin factor*-Fw 5′-CCCTGCTGCTGTTGCTTT-3′, *elongation factor*-Rv 5′-CCCATTTTGTCGGAGTGC-3′, *β-actin*-Fw 5′-TTGACAATGGATCCGGAATGT-3′, *β-actin*-Rv 5′-AAAACTGCCCTGGGTGCAT-3′.

### RNAi

Double-strand RNA (dsRNA) encoding *G. bimaculatus* AKHR and Hsl were prepared as described previously^17^. The target cDNAs for knockdown were amplified using the following primer sets containing the T7 promoter sequence at the 5′-end of each primer (underlined): T7-*AKHR*-Fw 5′-GCTTCTAATACGACTCACTATAGGTCAACCACATGCTCATGCAC-3′, T7-*AKHR*-Rv 5′-GCTTCTAATACGACTCACTATAGTCCAGCACATGAAGAAGACCAG-3′, T7-*Hsl*-Fw 5′-GCTTCTAATACGACTCACTATAGCAGTGCTGGTGTCGTTTGTG-3′ and T7-*Hsl*-Rv 5′-GCTTCTAATACGACTCACTATAGTCCACTGCTGGTTCCTTGTC-3′. DsRNA corresponding to nt 13–377 of the *DsRed2* (Clontech, Palo Alto, CA, USA) coding sequence was used as an experimental control. The following primers containing a 5′ T7 promoter sequence (underline) were used: T7-*DsRed2*-Fw 5′-GCTTCTAATACGACTCACTATAGAGAACGTCACCGAGTTCAT-3′ and T7-*DsRed2*-Rv 5′-GCTTCTAATACGACTCACTATAGCCGATGAACTTCACCTTGTAGA-3′. The dsRNAs were synthesized from 500 ng PCR products using T7 RNA polymerase according to the manufacturer’s instructions (TaKaRa, Shiga, Japan). The reaction mixture for RNA synthesis was treated with RQ DNase1 (Promega Co., Madison, WI, USA) and purified using phenol/chloroform extractions followed ethanol precipitation. The resulting RNAs were adjusted to 3 μg/μl in diethylpyrocarbonate-treated RNase-free water. The synthesized RNAs were denatured for 5 min at 100 °C and were annealed by cooling gradually to room temperature to produce dsRNAs. Knockdown was performed by injecting 6 μl of the dsRNA solution into the abdomen of fifth instar cricket nymphs one day after molting. RNAi efficiencies were determined by qRT-PCR using RNA extracted from the fat body of cricket nymphs 3 days after dsRNA treatment.

### Two choice assay

To analyze the dietary preference, we prepared two nutritionally different diets as described by Tsukamoto *et al*. with minor modification^41^. To normalize the dietary quantity, indigestible cellulose was mixed with two different nutrients (USFAs or SFAs), which were isocaloric (4 kj/g). All examined diets contained 33% casein and 33% dextrin for total energy. The USFA diet containing 15% oleic acid (18:1) and 15% linoleic acid (18:2), whereas the SFA diet consisted of 15% palmitic (16:0) acid and 15% stearic acid (18:0). The feeding frequency was represented by a heat map generated from 9 h continuous recorded video data as described by Fukumura *et al*.^42^. The Preference Index (PI) was defined by calculating the amount of USFA diet intake/the total amount of USFA and SFA diet intake × 100 (%) from 2 days post-dsRNA treatment for 3 days. The amount of food intake was determined as the dry weight by weighing the diet tablets before and after consumption. The diet tablets were dried by baking at 80 °C for 2 hours as described previously^41^.

### Statistical analyses

Statistical comparison of two groups was performed using Student’s *t*-test. Multiple comparisons were analyzed using Tukey’s test. *P*-values less than 0.05 were considered to be statistically significant.

## References

[1] Mayer RJ, Candy DJ (1969). Control of haemolymph lipid concentration during locust flight: An adipokinetic hormone from the corpora cardiaca. J. Insect Physiol..

[2] Beenakkers AMT (1965). Transport of fatty acids in *Locusta migratoria* during sustained flight. J. Insect Physiol..

[3] Arrese EL, Soulages JL (2010). Insect fat body: energy, metabolism, and regulation. Annu. Rev. Entomol..

[4] Canavoso LE, Jouni ZE, Karnas KJ, Pennington JE, Wells MA (2001). Fat metabolism in insects. Annu. Rev. Nutr..

[5] Gäde G, Hoffmann KH, Spring JH (1997). Hormonal regulation in insects: facts, gaps, and future directions. Physiol. Rev..

[6] Van der Horst DJ (2003). Insect adipokinetic hormones: release and integration of flight energy metabolism. Comp. Biochem. Physiol. B..

[7] Auerswald L, Siegert KJ, Gäde G (2005). Activation of triacylglycerol lipase in the fat body of a beetle by adipokinetic hormone. Insect Biochem. Mol. Biol..

[8] Auerswald L, Gäde G (2006). Endocrine control of TAG lipase in the fat body of the migratory locust. Locusta migratoria. Insect Biochem. Mol. Biol..

[9] Patel RT, Soulages JL, Arrese EL (2006). Adipokinetic hormone-induced mobilization of fat body triglyceride stores in *Manduca sexta*: Role of TG-lipase and lipid droplets. Arch. Insect Biochem. Physiol..

[10] Grönke S (2007). Dual lipolytic control of body fat storage and mobilization in. Drosophila. PLoS Biol..

[11] Arrese EL, Wells MA (1997). Adipokinetic hormone-induced lipolysis in the fat body of an insect, *Manduca sexta*: synthesis of sn-1, 2-diacylglycerols. J. Lipid Res..

[12] Gäde G, Simek P, Clark KD, Marco HG (2013). Five functional adipokinetic peptides expressed in the corpus cardiacum of the moth genus Hippotion (Lepidoptera, Sphingidae). Regul Pept.

[13] Bednářová A, Kodrík D, Krishnan N (2013). Adipokinetic hormone exerts its anti-oxidative effects using a conserved signal-transduction mechanism involving both PKC and cAMP by mobilizing extra- and intracellular Ca2+ stores. Comp. Biochem. Physiol. C Toxicol. Pharmacol..

[14] Noyes BE, Katz FN, Schaffer MH (1995). Identification and expression of the Drosophila adipokinetic hormone gene. Mol. Cell Endocrinol..

[15] Adamo SA, Roberts JL, Easy RH, Ross NW (2008). Competition between immune function and lipid transport for the protein apolipophorin III leads to stress-induced immunosuppression in crickets. J. Exp. Biol..

[16] Kaun KR, Chakaborty-Chatterjee M, Sokolowski MB (2008). Natural variation in plasticity of glucose homeostasis and food intake. J. Exp. Biol..

[17] Konuma T, Morooka N, Nagasawa H, Nagata S (2012). Knockdown of the adipokinetic hormone receptor increases feeding frequency in the two-spotted cricket *Gryllus bimaculatus*. Endocrinology.

[18] Kaufmann C, Merzendorfer H, Gäde G (2009). The adipokinetic hormone system in Culicinae (Diptera: Culicidae): Molecular identification and characterization of two adipokinetic hormone (AKH) precursors from *Aedes aegypti* and *Culex pipiens* and two putative AKH receptor variants from *A. aegypti*. Insect Biochem. Mol. Biol..

[19] Alves-Bezerra M (2016). Adipokinetic hormone receptor gene identification and its role in triacylglycerol metabolism in the blood-sucking insect *Rhodnius prolixus*. Insect Biochem. Mol. Biol..

[20] Huang JH, Bellés X, Lee HJ (2011). Functional characterization of hypertrehalosemic hormone receptor in relation to hemolymph trehalose and to oxidative stress in the cockroach *Blattella germanica*. Front. Endocrinol..

[21] Beenakkers AMT, Scheres JMJC (1971). Dietary lipids and lipid composition of the fat-body of *Locusta migratoria*. Insect Biochem..

[22] Van Dooremalen C, Ellers J (2010). A moderate change in temperature induces changes in fatty acid composition of storage and membrane lipids in a soil arthropod. J. Insect Physiol..

[23] Rozsypal J, Koštál V, Berková P, Zahradníčková H, Simek P (2014). Seasonal changes in the composition of storage and membrane lipids in overwintering larvae of the codling moth, *Cydia pomonella*. J. Therm. Biol..

[24] Lorenz MW, Anand AN (2004). Changes in the biochemical composition of fat body stores during adult development of female crickets, *Gryllus bimaculatus*. Arch. Insect. Biochem. Physiol..

[25] Gołębiowski M (2014). Adipokinetic hormone induces changes in the fat body lipid composition of the beetle *Zophobas atratus*. Peptides.

[26] Bharucha KN, Tarr P, Zipursky SL (2008). A glucagon-like endocrine pathway in D*rosophila* modulates both lipid and carbohydrate homeostasis. J. Exp. Biol..

[27] Bi J (2012). Opposite and redundant roles of the two *Drosophila* perilipins in lipid mobilization. J. Cell Sci..

[28] Raclot T (2003). Selective mobilization of fatty acids from adipose tissue triacylglycerols. Prog. Lipid Res..

[29] Gáliková M (2015). Energy homeostasis control in *Drosophila* adipokinetic hormone mutants. Genetics.

[30] Konuma T, Tsukamoto Y, Nagasawa H, Nagata S (2016). Imbalanced hemolymph lipid levels affect feeding motivation in the two-spotted cricket, *Gryllus bimaculatus*. PLoS One.

[31] Leyton J, Drury P, Crawford M (1987). Differential oxidation of saturated and unsaturated fatty acids *in vivo* in the rat. Br. J. Nutr..

[32] Grönke S (2005). Brummer lipase is an evolutionary conserved fat storage regulator in *Drosophila*. Cell Metab..

[33] Gavino VC, Gavino GR (1992). Adipose hormone-sensitive lipase preferentially releases polyunsaturated fatty acids from triglycerides. Lipids.

[34] Hazel JR, Sidell BD (2004). The substrate specificity of hormone-sensitive lipase from adipose tissue of the Antarctic fish *Trematomus newnesi*. J. Exp. Biol..

[35] Fougeron AS, Farine JP, Flaven-Pouchon J, Everaerts C, Ferveur JF (2011). Fatty-acid preference changes during development in *Drosophila melanogaster*. PLoS One.

[36] Wolesensky W, Joern A, Logan JD (2005). A model of digestion modulation in grasshoppers. Ecol. Modell..

[37] Lorenz MW (2003). Adipokinetic hormone inhibits the formation of energy stores and egg production in the cricket *Gryllus bimaculatus*. Comp. Biochem. Physiol. B Biochem. Mol. Biol..

[38] Zöllner N, Kirsch K (1962). The quantitative determination of lipids (micromethod) by means of the sulfo- phospho-vanillin reaction common to many natural lipids (all plasma lipids). Z Ges. Exp. Med..

[39] Yoshida T, Uetake A, Yamaguchi H, Nimura N, Kinoshita T (1988). New preparation method for 9-anthryldiazomethane (ADAM) as a fluorescent labeling reagent for fatty acids and derivatives. Anal. Biochem..

[40] Maekawa S (2015). Analysis of RNA decay factor mediated RNA stability contributions on RNA abundance. BMC Genomics.

[41] Tsukamoto Y, Kataoka H, Nagasawa H, Nagata S (2014). Mating changes the female dietary preference in the two-spotted cricket *Gryllus bimaculatus*. Front. Physiol..

[42] Fukumura K, Nagata S (2017). Behavioral tracing demonstrates dietary nutrient discrimination in two-spotted crickets G*ryllus bimaculatus*. Biosci. Biotech. Biochem..

